# Genome Report of Emergent Fish Pathogen *Edwardsiella piscicida* Recovered from *Pseudoplatystoma corruscans* in Brazil

**DOI:** 10.1128/mra.00796-22

**Published:** 2022-11-09

**Authors:** Natália A. Ferrari, João Vitor G. Takashe, Flávia F. Aburjaile, Vasco Azevedo, Mateus M. da Costa, Bertram Brenig, Francisco E. P. Rocha, Ulisses de P. Pereira

**Affiliations:** a Laboratory of Fish Bacteriology, Department of Preventive Veterinary Medicine, State University of Londrina, Londrina, Paraná, Brazil; b Preventive Veterinary Medicine Department, Veterinary School, Universidade Federal de Minas Gerais, Belo Horizonte, Minas Gerais, Brazil; c Laboratory of Cellular and Molecular Genetics, Institute of Biological Sciences, Federal University of Minas Gerais, Belo Horizonte, Minas Gerais, Brazil; d Universidade do Vale do São Francisco, Petrolina, Pernambuco, Brazil; e Department of Molecular Biology of Livestock, Institute of Veterinary Medicine, Georg August University Göttingen, Göttingen, Germany; Montana State University

## Abstract

Edwardsiella piscicida is a Gram-negative bacteria belonging to the *Hafniaceae* family which affects several species of marine and freshwater fish. We present the complete genome of *E*. *piscicida* strain BEP80 recovered from the Brazilian catfish named Surubim (Pseudoplatystoma corruscans), consisting a chromosome of 3,883,256 bp and no plasmids.

## ANNOUNCEMENT

The first description of the bacterial *Edwardsiella* genus occurred in 1960, with a characterization of the biochemical reactions for the species Edwardsiella tarda in *in vitro* tests (1). Retrospectively, the species Edwardsiella hoshinae and Edwardsiella ictaluri were also included at genus (2, 3). In recent years, the species *E. tarda* was found to comprise three phenotypically similar but genetically different taxa/genotypes, *E. tarda*, E. anguillarum, and E. piscicida, the last being more virulent for catfish than the others (4, 5). Until then, there had been no reports of *Edwardsiella piscicida* from fish in Brazil, so this is the first report of the strain deposited for this pathogen.

*Edwardsiella piscicida* strain BEP80, classified as a member of the *Gammaproteobacteria* of the order *Enterobacterales*, family *Hafniaceae*, and the genus *Edwardsiella*, was isolated at a farm with high mortality in Brazil and identified by seeding a cranial kidney fragment of a Surubim (Pseudoplatystoma corruscans) on a plate containing Mueller-Hinton agar (Kasvi, São José dos Pinhais, Brazil) enriched with 5% defibrinated sheep blood. After incubation at 29°C for 48 h, the colony was isolated and screening tests were executed (Gram stain, oxidase, and biochemical tests) to identify the bacterial genus. A single CFU was subjected to extraction of genomic DNA by use of a PureLink genomic DNA mini kit (Invotrogen, USA), according to the manufacturer's instructions. A multiplex PCR (mPCR) assay was performed to identify the bacterial species, which was characterized as *Edwardsiella piscicida* (6). For sequencing, the DNA was prepared using a NEBNext fast DNA fragmentation and library preparation kit (New England Biolabs, Inc.) sequenced on an Illumina HiSeq 2500 platform (2 × 150 bp), and FastQC v0.11.9 (7) was used to verify the sequencing quality (https://www.bioinformatics.babraham.ac.uk/projects/fastqc).

A total of 6,289,711 reads and coverage of 282× were generated. The genome assembly was performed using Unicycler v0.4.8 (8), which generated 119 contigs. Contigs smaller than 1,000 bp were removed, and the remaining contigs were submitted to CONTIGuator v2.7.4 software (9) to assemble the scaffold. *E. piscicida* strain CZ12001 was used as a reference (GenBank accession number CP090962.1), resulting in an initial scaffold. The genome was compared with other genomes of the same species, and gaps were curated with recursive rounds of short reads mapped using the Qiagen CLC Genomics Workbench v.22.1 (https://digitalinsights.qiagen.com/). The complete genome was annotated by the NCBI Prokaryotic Genome Annotation Pipeline (PGAP) and Rapid Annotation using System Technology v2.0 (RAST) (10) submitted to the GenBank database. The average nucleotide identity (ANI) (11) and digital DNA-DNA hybridization (dDDH) (12) estimations were determined using the Type (Strain) Genome Server (http://ggdc.dsmz.de/distcalc2.php).

The BEP80 genome consists of one circular chromosome of 3,883,256 bp with a G+C content of 59.7% and no plasmid. The circularization of the genome was accomplished based on the site of origin, using Ori-Finder 3 v1.0.1 software (13) (http://tubic.tju.edu.cn/Ori-Finder3/public/index.php). The absence of plasmids was confirmed using PlasmidFinder (14) (https://cge.cbs.dtu.dk/services/PlasmidFinder/). RAST annotation predicted 3,806 genes: 3,684 coding DNA sequences (CDS), 25 rRNA genes, and 97 tRNA genes. *E. piscicida* strain BEP80 shares ANIs of 99.45% (dDDH, 96.3%) with *E. piscicida* CZ12001 (CP090962.1), 94.92% (dDDH, 59.4%) with *E. anguillarum* ET080813 (CP006664.1), 92.45% (dDDH, 48.2%) with *E. ictaluri* MS-17-156 (CP028813.1), 83.54% (dDDH, 24.7%) with *E. hoshinae* FDAARGOS 940 (CP065626.1), and 83.93% (dDDH, 25.4%) with *E. tarda* Kc-Pc-HB1 (CP023706.1).

### Data availability.

The complete genome sequence of *Edwardsiella piscicida* BEP80 has been deposited in GenBank under accession number CP101126.1, BioProject identifier PRJNA856875, BioSample identifier SAMN29594514, and Sequence Read Archive (SRA) identifier SRR21788775.

## References

[1] Ewing WH, McWhorter AC, Escobar MR, Lubin AH. 1965. Edwardsiella, a new genus of Enterobacteriaceae based on a new species, E. tarda. Int J Syst Evol Microbiol 15:33.

[2] Grimont PAD, Grimont F, Richard C, Sakazaki R. 1980. Edwardsiella hoshinae, a new species of Enterobacteriaceae. Curr Microbiol 4:347–351. doi:10.1007/BF02605375.

[3] Hawke JP, McWhorter AC, Steigerwalt AG, Brenner DJ. 1981. Edwardsiella ictaluri sp. nov., the causative agent of enteric septicemia of catfish. Int J Syst Bacteriol 31:396–400. doi:10.1099/00207713-31-4-396.

[4] Abayneh T, Colquhoun DJ, Sørum H. 2013. Edwardsiella piscicida sp. nov., a novel species pathogenic to fish. J Appl Microbiol 114:644–654. doi:10.1111/jam.12080. 23167785

[5] Shao S, Lai Q, Liu Q, Wu H, Xiao J, Shao Z, Wang Q, Zhang Y. 2015. Phylogenomics characterization of a highly virulent Edwardsiella strain ET080813^T^ encoding two distinct T3SS and three T6SS gene clusters: propose a novel species as Edwardsiella anguillarum sp. nov. Syst Appl Microbiol 38:36–47. doi:10.1016/j.syapm.2014.10.008. 25466920

[6] da Costa AR, Chideroli RT, Lanes GC, Ferrari NA, Chicoski LM, Batista CE, Pandolfi VCF, Ware C, Griffin MJ, Dos Santos AR, de Carvalho Azevedo VA, da Costa MM, de Pádua Pereira U. 2022. Multiplex PCR assay for correct identification of the fish pathogenic species of Edwardsiella genus reveals the presence of E. anguillarum in South America in strains previously characterized as E. tarda. J Appl Microbiol 132:4225–4235. doi:10.1111/jam.15538. 35332638

[7] Babraham Bioinformatics. 2022. FastQC: a quality control tool for high throughput sequence data. https://www.bioinformatics.babraham.ac.uk/projects/fastqc/. Retrieved 10 October 2022.

[8] Wick RR, Judd LM, Gorrie CL, Holt KE. 2017. Unicycler: resolving bacterial genome assemblies from short and long sequencing reads. PLoS Comput Biol 13:e1005595. doi:10.1371/journal.pcbi.1005595. 28594827 PMC5481147

[9] Galardini M, Biondi EG, Bazzicalupo M, Mengoni A. 2011. CONTIGuator: a bacterial genomes finishing tool for structural insights on draft genomes. Source Code Biol Med 6:11. doi:10.1186/1751-0473-6-11. 21693004 PMC3133546

[10] Aziz RK, Bartels D, Best A, DeJongh M, Disz T, Edwards RA, Formsma K, Gerdes S, Glass EM, Kubal M, Meyer F, Olsen GJ, Olson R, Osterman AL, Overbeek RA, McNeil LK, Paarmann D, Paczian T, Parrello B, Pusch GD, Reich C, Stevens R, Vassieva O, Vonstein V, Wilke A, Zagnitko O. 2008. The RAST Server: rapid annotations using subsystems technology. BMC Genomics 9:75. doi:10.1186/1471-2164-9-75. 18261238 PMC2265698

[11] Arkin AP, Cottingham RW, Henry CS, Harris NL, Stevens RL, Maslov S, Dehal P, Ware D, Perez F, Canon S, Sneddon MW, Henderson ML, Riehl WJ, Murphy-Olson D, Chan SY, Kamimura RT, Kumari S, Drake MM, Brettin TS, Glass EM, Chivian D, Gunter D, Weston DJ, Allen BH, Baumohl J, Best AA, Bowen B, Brenner SE, Bun CC, Chandonia JM, Chia JM, Colasanti R, Conrad N, Davis JJ, Davison BH, Dejongh M, Devoid S, Dietrich E, Dubchak I, Edirisinghe JN, Fang G, Faria JP, Frybarger PM, Gerlach W, Gerstein M, Greiner A, Gurtowski J, Haun HL, He F, Jain R, et al. 2018. KBase: the United States Department of Energy systems biology knowledgebase. Nat Biotechnol 36:566–569. doi:10.1038/nbt.4163. 29979655 PMC6870991

[12] Goris J, Konstantinidis KT, Klappenbach JA, Coenye T, Vandamme P, Tiedje JM. 2007. DNA-DNA hybridization values and their relationship to whole-genome sequence similarities. Int J Syst Evol Microbiol 57:81–91. doi:10.1099/ijs.0.64483-0. 17220447

[13] Wang D, Lai F-L, Gao F. 2021. Ori-Finder 3: a web server for genome-wide prediction of replication origins in Saccharomyces cerevisiae. Brief Bioinform 22:bbaa182. doi:10.1093/bib/bbaa182. 34020544

[14] Carattoli A, Zankari E, Garciá-Fernández A, Larsen MV, Lund O, Villa L, Aarestrup FM, Hasman H. 2014. In silico detection and typing of plasmids using PlasmidFinder and plasmid multilocus sequence typing. Antimicrob Agents Chemother 58:3895–3903. doi:10.1128/AAC.02412-14. 24777092 PMC4068535

